# Revisiting the effectiveness of cognitive‐behavioural therapy for reducing reoffending in the criminal justice system: A systematic review

**DOI:** 10.1002/cl2.1425

**Published:** 2024-07-31

**Authors:** Andrew Smith, Anton Roberts, Karolina Krzemieniewska‐Nandwani, Liz Eggins, Will Cook, Chris Fox, Shadd Maruna, Stephanie Wallace, Kirstine Szifris

**Affiliations:** ^1^ National Foundation for Education Research Slough UK; ^2^ Manchester Metropolitan University Manchester UK; ^3^ The University of Queensland Brisbane Queensland Australia; ^4^ Queen's University Belfast Belfast UK; ^5^ Lancaster University Lancaster UK

**Keywords:** CBT, cognitive behavioural therapy, criminal justice, recidivism, reoffending

## Abstract

This is the protocol for a Campbell systematic review. The objectives are as follows. The proposed systematic review is an update to, and extension of, Lipsey et al. (2007). As such we build on their previous aims to: (i) Assess and synthesise the overall impact of cognitive behavioural therapy (CBT) on offender recidivism; (ii) Examine possible sources of variability in the effectiveness of CBT. Data permitting, we will examine if the effectiveness of CBT varies by: (a) Characteristics of the CBT intervention (e.g., cognitive restructuring vs. cognitive skills training, group v. individual implementation; and/or custodial v. community setting, and/or), (b) Characteristics of the population (e.g., juveniles vs. adult offenders), (c) Implementation factors (e.g., implementing practitioner, use of structured/manualised approaches, delivery mode, and/or programme duration or intensity), (d) Evaluation methods (e.g., randomised vs. non‐randomised research designs); (iv) Determine whether there is a decline in the effect of CBT on recidivism over time; and (v) Investigate whether there is an interaction between implementation factors and time in terms of the effect on recidivism.

## 1 BACKGROUND

### 1.1 The problem, condition, or issue

Cognitive behavioural therapy (CBT) has assumed a central role in criminal justice interventions (Brooker & Gojkovic, 2009) on the grounds that a strong foundation of international evidence, including previous systematic reviews and meta‐analyses, demonstrates its effectiveness in reducing recidivism (e.g., Armelius & Andreassen, 2007; Feucht & Holt, 2016; Henwood, 2015; Lipsey et al., 2007; Usher & Stewart, 2012). In England and Wales, for example, most accredited programmes delivered in the Criminal Justice System (CJS) are rooted in cognitive‐behavioural methodologies and aim to change the attitudes and thinking of people convicted of an offence (Harvey & Smedley, 2012). However, to gain accreditation in England and Wales, programmes primarily have to demonstrate that they follow principles associated with the Risk, Needs, Responsivity (RNR) model (Andrews & Bonta, 2006), so the evidence for the effectiveness of many accredited programmes is one step removed from the programmes themselves.

This might be of little concern if the broader evidence about CBT was uncontested. However, questions about the efficacy of CBT outside of the CJS, as well as concerns about the design and delivery of cognitive‐behavioural programmes within the CJS are growing (Kendall & Mair, 2004). For example, a recent review of one‐to‐one CBT for the treatment of depression (Johnsen & Friborg, 2015) found its impact had fallen substantially and linearly over time, from early trials in the late 1970s to contemporary trials. Similar findings were presented by Friborg and Johnsen (2017), although the nature of the decline was shown to be non‐linear. Results from a subsequent study using a similar design to review temporal changes in the effects of group CBT as a treatment for depression was less clear‐cut (Johnsen & Thimm, 2018) and a more recent study of mindfulness‐based cognitive therapy (Thimm & Johnsen, 2020) did not find a decline effect. Johnson and Friborg's 2015 finding has also been questioned and reassessed (Cristea et al., 2017; Ljótsson et al., 2017), leading to different conclusions to the original authors about the nature of the decline.

There is a myriad of explanations for the potential decline in CBT's effectiveness over time, for example, researcher allegiance, the established effect of initial researchers tending to find stronger effect sizes than later ones. Many scholars have addressed the risk of treatment drift, due to the proliferation of CBT as an apparent panacea intervention, leading to a risk of lower treatment fidelity (Waller, 2009). Waltman et al. (2016) in addressing some of the limitations of the Johnsen and Friborg meta‐analysis, while expressing criticism of the methods employed, acknowledged the risk of model change – ‘Therefore, Johnsen and Friborg's meta‐analysis may, in fact, herald a call for a re‐emphasis of quality, fidelity, and competence in the practice of CBT’ (p. 2). It is clear therefore that the question of a potential decline effect of CBT over time is not resolved, and this is not something which has been studied in cognitive‐behavioural programmes aimed at reducing recidivism.

Furthermore, CBT‐related effect sizes may be attenuated by implementation factors, for example, Johnsen and Thimm (2018) noted that in trials conducted without following a set manual, effect sizes increased over time. Research into psychological treatments for offending has highlighted the role of staff and programme implementation factors as moderators of programme outcomes (Gannon et al., 2019). In standard therapeutic practice, success is predicated on practitioners receiving adequate training and supervision, and variations in competence levels of prison staff may be particularly relevant in the CJS and its use of CBT (Brosan et al., 2007). These findings are important and highly relevant, as the use of standardised (i.e., manualised) programmes, using less qualified staff, is a delivery model which is routinely used in the CJS internationally. These findings echo concerns about the use of cognitive‐behavioural programmes in the CJS, specifically ‘sausage factory’ interventions (Hobbs & Cook Consulting, 2001) and unresponsive ‘one size fits all’ approaches to rehabilitation and learning (Clarke & Wydall, 2004). A substantial body of evaluation research also supports the idea of a ‘scale‐up penalty’ in the delivery of criminal justice interventions, whereby measures of effectiveness drop considerably when an intervention moves from a demonstration project to large‐scale delivery across a service (Yohros & Welsh, 2019).

The aim of this review is therefore to revisit the effectiveness of cognitive‐behavioural programmes in the CJS, updating and extending the previous systematic review and meta‐analysis undertaken by Professor Mark Lipsey and colleagues (2007), principally to investigate whether the effects of cognitive‐behavioural programmes are decreasing over time in the CJS, and how implementation factors influence the effects observed.

### 1.2 The intervention

As an intervention, cognitive behaviour therapy has a broad range of treatment variations, although at its most rudimentary, it emphasises the role of dysfunctional thinking as the primary factor to all psychological disturbances (Beck, 2021). As a practice it explores and questions the validity of thoughts, beliefs, and maladaptive behaviours, and offers a range of solutions such as exposure and cognitive homework exercises. Cognitive‐behavioural treatment approaches have been developed and implemented in various settings worldwide since the 1960s.

CBT as it is employed in the CJS is often implemented with the aim of reducing reoffending rather than reducing psychological distress, generally in the form of a group‐based structured cognitive‐behavioural programmes (Tafrate & Mitchell, 2014). In line with psychotherapeutic applications of CBT, which associate emotional distress and behavioural problems with maladaptive thinking, its application in criminal justice settings is based on the premise that offending behaviours are motivated by criminogenic thinking patterns (see Section 1.3).

Lipsey and colleagues used the term ‘brand name CBT programmes’ to identify commonly used examples such as Reasoning and Rehabilitation, Moral Reconation Therapy, Aggression Replacement Training, the Thinking for a Change curriculum, and the Cognitive Interventions Programme. Since publishing their review in 2007 other programmes have been developed and implemented in CJSs worldwide. Taking England and Wales as an example, some of these include Control of Violence for Angry Impulsive Drinkers (COVAID), Building Better Relationships, and the Thinking Skills Programme. The latter is ‘a cognitive skills programme which addresses the way offenders think and their behaviour associated with offending. The programme aims to reduce reoffending by engaging and motivating, coaching, and responding to individual need and building on continuity. It supports offenders developing skills in setting goals and making plans to achieve these without offending’ (Ministry of Justice, 2021, p. 2).

Hayes and Hofmann (2017) argue that the development of CBT, rather than being monolithic, has gone through distinct ‘eras, generations, or waves’ (p. 245). The focus of Lipsey et al.'s (2007) review on classic (first/second‐wave) CBT is a natural consequence of the time at which their work was undertaken and published. A ‘third wave’ of CBT was however heralded in 2004 (Hayes & Hofmann, 2017), and the current review will also (in addition to waves one and two) incorporate evidence of the efficacy of interventions which include such approaches in the criminal justice settings. It may be the case, that searches undertaken by Lipsey et al. did not find instances of these types of CBT variants within the CJS before their published review. Third wave models include Acceptance and Commitment Therapy (ACT), Dialectical Behaviour Therapy (DBT), Mindfulness‐Based Cognitive Therapy (MCBT), Metacognitive Therapy (MCT) and others, variously emphasising ‘mindfulness, emotions, acceptance the relationship, values, goals, and meta‐cognition’ (Hayes & Hofmann, 2017, p. 245). In practice the ‘waves’ of CBT do not have discrete boundaries that are easily defined into pure ‘waves’. These distinctions are primarily a conceptual framework, and the researchers accept there will inevitably be a degree blurring of the lines between them, however they do provide a useful overall picture of CBT's development (see Supporting Information: Appendix C for wave classification). The wave typology can also be utilised to aid in the CBT classification/explanation during the screening process. Whilst these third wave approaches are relatively new and therefore not as prevalent in CJSs compared to the more well established second wave based programmes, there is some recent evidence of their use (e.g., Murray et al., 2018).

### 1.3 How the intervention might work

The use of cognitive‐behavioural programmes in the CJS is rooted in the RNR model of rehabilitation developed in the early 1990s by Andrews and Bonta (2006). The RNR model was developed as a psychological perspective of criminal conduct which outlined a General Personality and Cognitive Social Learning Theory of Criminal Conduct (Andrews & Bonta, 2006). This perspective highlighted what is sometimes referred to as an ‘antisocial personality pattern’ and the role of cognition (i.e., self‐regulation, deliberate rational decision making, and attitudes values and beliefs) in criminogenic thinking patterns and criminal behaviour (see Bonta & Andrews, 2007, for an overview).

The focus of the RNR models of rehabilitation is on reducing risk of reoffending (as opposed to punishment or deterrence issues; Hayward, 2009). Within this model, Andrews and Bonta (2006) highlighted eight central risk factors including ‘procriminal attitudes’ relating to thoughts, values, and sentiments supportive of criminal conduct (Bonta & Wormith, 2007). This suggests that a person's ‘criminogenic needs’ are those risk factors that are dynamic (i.e., changeable) and specifically relate to chances of recidivism.

With the emphasis on thinking and attitudes within the RNR model of criminal conduct, cognitive‐behavioural approaches to rehabilitation took favour. More specifically,

…rather than attempting to change the whole personality or circumstances of an offender, cognitive behavioural programmes focus on specific unacceptable behaviours and seek to modify these by correcting distortions in the way offenders think about their crimes. (Hayward, 2009, p. 142).

Cognitive‐behavioural treatment is therefore directed toward changing offenders' distorted or dysfunctional cognitions, which they may experience as verbal or pictorial events in the stream of consciousness, cognitive schemas, thinking, conceptualisations, perceptions, reflections, beliefs, rules, or automatic thoughts (Lipsey & Landenberger, 2006).

The research around exactly how CBT is understood to reduce recidivism is less developed than that which demonstrates its effectiveness. Its mechanism of action (and justification of its use) is arguably that:

A: A key risk factor (and dynamic criminogenic need) in the psychology of criminal conduct relates to the thinking and attitudes of offenders.
B: CBT is a mode of therapy that addresses and changes the thinking and attitudes of the recipients.
C: Therefore cognitive‐behavioural approaches can be used to reduce risk of reoffending.

Whilst this makes some logical sense, we note that the type of thinking and attitudes addressed by CBT in a therapeutic setting are somewhat distinct from those addressed in a criminal justice setting. In particular, CBT outside of criminal justice is used to address phobias, anxiety, and depressive thoughts (Padesky & Beck, 2003) whilst within the CJS it addresses cognitive distortions that support offending behaviour. For example, CBT is used widely with people convicted of sexual offences and may address belief systems that relate to offence‐related beliefs (e.g., that a victim desires sex), or minimisation of personal responsibility (Schaffer et al., 2010). There is, therefore, an inferential leap to assume that a treatment that addresses depressive thoughts can address criminogenic thoughts.

### 1.4 Why it is important to do this review

Lipsey and colleagues' (2007) review, one of The Campbell Collaboration's most cited systematic reviews (Wiley, 2021), is now approaching 15 years old, and has not been updated by the original authors since publication. The use of cognitive‐behavioural programmes in CJSs around the world has increased since then and a third wave of CBT has started to evolve and inform a new generation of interventions.

This review will therefore update and Lipsey et al.'s work to:

(i) incorporate studies undertaken and published to 2024;
(ii) incorporate more recent developments in the application of cognitive‐behavioural programmes within the CJS, including mindfulness‐based and other third wave approaches (see Supporting Information: Appendix C).

In addition to this update, we will extend the original review by:

(i) investigating whether the efficacy of cognitive‐behavioural programmes in the CJS is diminishing over time. Given some of the aforementioned critiques of CBT in the CJS, and research in other areas which identifies a possible reduction in the effects of CBT over time, it is a good opportunity to investigate whether there is any evidence for similar decline effects (e.g., by year of study, or year of publication);
(ii) Considering the role of implementation factors (e.g., staff training, manualised approaches) and their influence on programme effects.

The review is one work package within a wider project with an established Advisory Board, which seeks to influence local and national policy and commissioning practice in relation to the use of CBT in England and Wales. The findings of the review will be synthesised with findings from the parallel work package (a review of the theory and implementation of CBT in the CJS) to consider implications for the use of cognitive‐behavioural programmes in the English and Welsh CJS. Part of the importance of this review is that this project will provide empirical evidence to speak to some of the wider criticisms and concerns and provide clear evidence for recommendations for policy and practice concerning the use of cognitive‐behavioural programmes in CJSs around the world.

## 2 OBJECTIVES

The proposed systematic review is an update to, and extension of, Lipsey et al. (2007). As such we build on their previous aims to:

1. Assess and synthesise the overall impact of CBT on offender recidivism.
2. Examine possible sources of variability in the effectiveness of CBT. Data permitting, we will examine if the effectiveness of CBT varies by:
  a. Characteristics of the CBT intervention (e.g., cognitive restructuring vs. cognitive skills training, group v. individual implementation; and/or custodial v. community setting, and/or)
  b. Characteristics of the population (e.g., juveniles vs. adult offenders).
  c. Implementation factors (e.g., implementing practitioner, use of structured/manualised approaches, delivery mode, and/or programme duration or intensity).
  d. Evaluation methods (e.g., randomised vs. non‐randomised research designs).
3. Determine whether there is a decline in the effect of CBT on recidivism over time.
4. Investigate whether there is an interaction between implementation factors and time in terms of the effect on recidivism.

## 3 METHODS

### 3.1 Criteria for considering studies for this review

#### 3.1.1 Types of studies

We will include randomised controlled trials, exclusively those that focus on the individual as the unit of allocation, and robust quasi‐experimental designs. Studies with robust quasi‐experimental designs must be those that either exploit credible exogenous variation (regression discontinuity; difference‐in‐difference; natural experiment), those with a well‐matched comparison group (i.e., propensity score or statistically matched) or those that utilise both a pre‐ and post‐intervention measure of the outcome. For studies using matching, this must be on demographic or criminological variables such as gender or sex, ethnicity, age, risk level, offence type, and/or offending history. Studies with multiple treatment arms will be included, providing the above criteria are met. We will only include studies involving a comparison group such that subjects receiving treatment are compared with those in a control condition. Control conditions may be ‘no treatment’, ‘treatment as usual’, ‘usual care or supervision’, or ‘waitlist’.

In line with Lipsey et al. (2007), we will search for studies published 1965 or later (although we note that their review did not find any studies before 1980). Studies can be undertaken anywhere in the world, though must be published in English. The restriction to English‐language publications will be to make effective use of the resources of the review team.

#### 3.1.2 Types of participants

Only studies of convicted offenders – juveniles (10–17 years) or adults (≥18 years) – will be included. Where it is not clear whether the offender has been convicted, offenders will be included if it is clear that they were recipients of the intervention as a result of contact with the CJS (i.e., as per Lipsey et al., 2007; ‘treated while on probation, incarcerated/institutionalized, or during aftercare/parole’). There are no restrictions participant ethnicity or sex/gender. If a study includes both eligible and ineligible participants, we will try to extract an effect size for the eligible group. If this is not possible using the published text (and in cases where >50% of the sample are eligible), we will contact the study authors to request outcome data for the eligible participants. If this data cannot be sourced, we will exclude the study from the review.

Lipsey et al. (2007) only included studies of offenders drawn from a general offender population rather than those being treated for specific offences (e.g., sex offences) or behaviours (e.g., drug use). Our review will include those that focus on the general offender population while still excluding special groups such as sexual offenders and vulnerable prisoners. All the offence types of the participants are included with the notable exception of sexual offences.

#### 3.1.3 Types of interventions

Consistent with Lipsey et al. (2007), our review will include studies investigating any variant of CBT. This can either be a recognised programme (e.g., an ‘accredited’ programme) or another cognitive‐behavioural programme that is directed toward cognitive restructuring, teaching new cognitive skills, or that involves therapeutic techniques associated with CBT. A cognitive‐behavioural intervention is often offered as one component of a multi‐model programme (e.g., alongside education and training before release from prison). Where this is the case, the cognitive‐behavioural component must have been provided to all participants and constitute a major focus of the programme. Specifically, we will require that the cognitive‐behavioural component constitutes more than 50% of the delivery time of the programme. In circumstances where it is difficult to ascertain the relative percentage of the CBT intervention component, such studies will be flagged for additional review/adjudication by the entire research team, and the authors of the study will be contacted for clarification on the relative weight of interventions. Where the relative percentage cannot be ascertained, we will include the study but conduct sensitivity analysis based on the level of CBT dosage and the exclusion/inclusion of these studies.

We will include interventions based on cognitive behavioural principles or the following third‐wave therapies, including: ACT, DBT, MBCT, MCT (see Supporting Information: Appendix C for CBT wave classification). We expect that the overwhelming majority of programmes will be delivered to a group, but we will also include programmes delivered to individuals which are identified by our searches.

We define cognitive‐behavioural principles in a manner consistent with Lipsey and Landenberger (2006, p. 14):

A. Cognitive‐behavioral treatment is directed toward changing offenders' distorted or dysfunctional cognitions (verbal or pictorial events in stream of consciousness, cognitive schemas, thinking, conceptualizations, perceptions, reflections, beliefs, rules, automatic thoughts) OR teaching new cognitive skills in areas where offenders show deficits with the expectation that such cognitive changes will result in more adaptive and/or less antisocial behavioral responses. NOTE: Behavioral or training approaches to improving social skills are only eligible if they clearly emphasize cognitive variables as the mediators of social skills, e.g., use of rewards and punishments to shape social behavior directly would not be eligible.

B. The therapeutic activities consist of specific, relatively structured learning experiences designed to affect such cognitive processes as monitoring thoughts, recognizing connections between cognition, affect, and behavior, examining evidence for and against thoughts, substituting reality‐oriented interpretations for biased ones, identifying, and altering dysfunctional beliefs, and the like. Examples of such techniques include: problem solving or decision‐making exercises, hassle logs, monitoring behavior/thoughts, rational responding to ‘risky’ thoughts, behavioral experiments, distraction and refocusing, guided imagery, and self‐statement logs.

We will *exclude* interventions which are evaluations of approaches to offender supervision or case management which incorporate CBT skills as an aspect of supervision (e.g., core correctional skills, STARR – Staff Training Aimed at Reducing Re‐arrest). This is because the relative weighting of the CBT and offender supervision components would be highly difficult to differentiate. Additionally, typically supervision/case management is primarily concerned with the management of risk and assessment of the criminogenic needs of offenders which is arguably distinct from the CBT's treatment principles. We will also exclude mindfulness‐based interventions which do not use cognitive‐behavioural methods (e.g., mindfulness only interventions).

#### 3.1.4 Types of outcome measures

We will include studies where the outcome is a measure of recidivism, which we define as official measures of rearrest, reconvictions (binary, frequency, severity), and/or breaches of condition (e.g., recalls to custody or return to court). We will include both outcomes measured from official sources and those from self reported measures where the outcome is reported by the offender. The study must report a quantitative measure of reoffending as an outcome variable, with enough detail that we can calculate an effect size from the included information. Where there is not enough detail we will attempt where possible to contact study authors for clarification.

#### 3.1.5 Duration of follow‐up

Measurement of recidivism at 12 months posttreatment is the basis for reoffending statistics in the United Kingdom, but previous reviews in this field (e.g., Smith et al., 2018) have identified studies measuring outcomes over longer terms. However, for the purposes of this review we will consider all follow up periods.

#### 3.1.6 Types of settings

We will include studies from any custodial (e.g., prisons) or community settings (e.g., where participants are on probation/parole or serving community sentences).

### 3.2 Search methods for identification of studies

#### 3.2.1 Electronic searches

We will undertake a search of the following electronic databases available through our externally recruited Information Specialist's university libraries.

**Table:** 

Cochrane	Cochrane Central Register of Controlled Trials (CENTRAL) Cochrane Database of Systematic Reviews
EBSCOhost	Criminal Justice Abstracts Cumulative Index to Nursing and Allied Health Literature (CINAHL) ERIC
Elsevier	Embase Scopus
Ovid	Joanna Briggs Institute EBP Database Medline PsycEXTRA PsycINFO
ProQuest	Applied Social Sciences Index & Abstracts Criminal Justice Database Dissertations and Theses Global International Bibliography of the Social Sciences Nursing and Allied Health Database Psychology Journals PTSDPubs Public Health Database Social Science Database Social Services Abstracts Sociological Abstracts Sociology Database
Web of Science	Book Citation Index – Social Sciences & Humanities Emerging Sources Citation Index (ESCI) Conference Proceedings Citation Index – Social Science & Humanities Social Science Citation Index
Other	*Campbell Systematic Reviews* journal (Wiley) Health Technology Database (via https://database.inahta.org) Database of Abstracts of Reviews of Effectiveness (via https://www.crd.york.ac.uk/CRDWeb/) National Criminal Justice Reference Service (NCJRS) Abstract Database

The search will be for studies conducted anywhere in the world, published in English from 1965 to date. We note that Lipsey et al. (2007) did not find any studies before 1980, but we will search from the earlier year to replicate their approach and to ensure that no relevant studies are missed on account of the date. Studies will be included regardless of publication status.

In consultation with an Information Retrieval specialist from the Crime and Justice Coordinating Group (Elizabeth Eggins), we have developed and tested the search strategy provided in Supporting Information: Appendix A. The search terms and structure were developed and tested using an iterative approach using database thesauri and indexes and by examining the citation and terminology characteristics of studies included in existing reviews of CBT and closely related intervention models.

#### 3.2.2 Searching other resources

We will also undertake an electronic search of websites of relevant governmental agencies and organisations. The following websites will be searched for reports and other grey literature:

**Table:** 

Organisation	URL
Australian Institute of Criminology	https://www.aic.gov.au
Crime Reduction Toolkit	https://www.college.police.uk/research/crime-reduction-toolkit
CrimeSolutions	!https://crimesolutions.ojp.gov
DART‐Europe E‐theses Portal	https://www.dart-europe.org/basic-search.php
Evidence for Policy and Practice Information & Coordinating Centre (EPPI‐Centre)	https://eppi.ioe.ac.uk/cms/
Home Office, UK (including Ministry of Justice and Justice Data Lab)	https://www.gov.uk/government/organisations/home-office
Jill Dando Institute of Security and Crime Science	https://www.ucl.ac.uk/jill-dando-institute/ucl-jill-dando-institute-security-and-crime-science
Justice Research and Statistics Association	https://www.jrsa.org
National Council for Crime Prevention, Sweden	https://www.government.se/government-agencies/the-swedish-national-council-for-crime-prevention/
National Institute of Justice (United States)	https://nij.ojp.gov
National Institute of Corrections (United States)	https://nicic.gov
Networked Digital Library of Theses and Dissertations	https://ndltd.org
Office of Juvenile Justice and Delinquency Prevention	https://ojjdp.ojp.gov
RAND Corporation	https://www.rand.org
Social Care Institute for Excellence (SCIE, including ELSC and Social Care Online)	https://www.scie.org.uk
Social Science Research Network	https://www.ssrn.com/index.cfm/en/
The Urban Institute	https://www.urban.org
Washington State Institute for Public Policy	https://www.wsipp.wa.gov

We will also conduct a series of supplementary search steps. First, we will hand search the following key journals, examining the issues for the year before the date of the electronic database searches:

- *Crime & Delinquency*
- *Criminal Justice Policy Review*
- *Criminology*
- *Criminology & Public Policy*
- *International Journal of Offender Therapy and Comparative Criminology*
- *Journal of Contemporary Criminal Justice*
- *Journal of Experimental Criminology*
- *Journal of Forensic Practice*
- *Probation Journal*

Second, we will contact leading experts in the field to request unpublished studies and will incorporate studies from electronic databases supplied by Professor Lipsey in July 2021. Third, we will review reference lists for all included studies and existing systematic reviews for additional potentially eligible studies. Fourth, we will conduct forward citation searches on all included studies using Google Scholar.

### 3.3 Data collection and analysis

#### 3.3.1 Description of methods used in primary research

Our search strategy will explicitly search for randomised and quasi‐experimental designs, although we are aware that evaluations in criminal justice can take a wider range of forms. An example type of study that would be included in the review is by Khodayarifard et al. (2010) who conducted a multiple arm randomised trial of group‐based CBT for male prisoners in Iran, using an individual intervention treatment arm, a group intervention treatment arm, and a no‐treatment control group. The intervention was named R&R, and this version of CBT was focused on treating prisoners with mental disorders. Effectiveness was evaluated on the basis of recidivism after 1 year of release from prison.

Based on the original review by Lipsey and colleagues, we anticipate that the majority of studies will be implemented in the United States, will focus on adults, and will use a measure of recidivism based on a set follow up period after the intervention. We also anticipate that rearrest to be the most common type of outcome measure and that the setting of the intervention will be approximately evenly distributed across custodial and community settings.

#### 3.3.2 Criteria for determination of independent findings

We will review all included studies to identify multiple reports of single studies. Multiple reports of the same study will be linked under a ‘parent study’ in review management software (DistillerSR) and all reports will be used on data extraction. However, each study will only contribute one conceptually distinct outcome in each analysis. Given the nature of the outcome inclusion criteria, we do not anticipate that studies will report more than one conceptually similar measure of an eligible outcome which would then necessitate statistically combining outcomes into a single effect size for a study.

#### 3.3.3 Selection of studies

The de‐duplicated results of the systematic search will be imported into *DistillerSR* review management software (Evidence Partners, 2022). The first stage of screening will comprise title/abstract screening and will use the ‘two to exclude’ setting. This means that two independent screeners will need to exclude a record, whereas only one screener will need to include the record. Before screening begins, standardised screening guides will be provided to all screeners and each screener will then screen the same set of 25 records before beginning formal screening on the systematic search results. Further training and guidance will be provided if the results of the screening test‐set suggest inadequate understanding of the screening protocol, which we define as having no false negative decisions (i.e., incorrect exclusions). At this stage of study selection, screeners will be first asked if they are including or excluding the record. If indicating that they will exclude the record, they will be prompted to select a reason from a list of reasons: (1) ineligible document type (e.g., book review); (2) duplicate record; (3) not an empirical study focused on CBT; or (4) not an empirical study using juvenile or adult participants involved in the CJS. A third review author will resolve any studies for which screening decisions are unclear or cannot be determined. Although the lead reviewer will have the final decision, all study authors will then meet to discuss any disagreements/ineligibilities regarding the studies.

All studies that pass the title and abstract screening stage will then move on to final screening stage using the full‐text document, with the same screening setting in *DistillerSR* (i.e., ‘two to exclude’) and training procedure used for the title and abstract screening stage. For records where a full‐text document cannot be sourced through authors' institutional libraries, an attempt will be made to screen the record using the title and/or abstract. Where a decision cannot be made based on the title and/or abstract, we will contact the first author of the document to either provide a full‐text document or to verify eligibility for the review. At this stage of study selection, screeners will be first asked if they are including or excluding the record. If indicating that they will exclude the record, they will be prompted to select a reason from a list of reasons: (1) ineligible document type (e.g., book review); (2) duplicate record; (3) not an evaluation of an eligible CBT intervention; (3) ineligible population; (4) ineligible research design; and (5) ineligible outcome. Where a record/study cannot be excluded with certainty, the reference will be included in the ‘*Studies awaiting classification*’ reference list in the final review.

*DistillerSR* utilises artificial intelligence and machine learning to prioritise citations and abstracts based on the probability of eligibility, informed by prior human decisions. This function streamlines the systematic review process by presenting records (citations and abstracts) to screeners in order of their likelihood of inclusion. The software continuously estimates the percentage of potentially eligible records identified as screening progresses. This provides the option for authors to stop screening when a particular threshold has been reached, without humans necessarily needing to screen all records captured by the systematic search. Ranked title and abstract screening will continue until DistillerSR estimates that 95 percent of the potentially eligible studies have been identified. Once this threshold has been reached, iterative sets of 50 randomly ordered titles and abstracts (records) will be screened until a set of 50 records contains no potentially eligible records. Once this point is reached at the title and abstract screening stage, the remaining unscreened records will be treated as exclusions. At the full‐text screening stage, all records (documents) will be screened by humans.

#### 3.3.4 Data extraction and management

A standardised, pre‐piloted form will be used to extract data from the included studies. One review author will extract data, and a second researcher will review 25% of data extracted. A senior reviewer will assist with any discrepancies and to clarify any ambiguities. Required data that cannot be extracted from the published reports will be requested from study authors. The data extraction form is included in Supporting Information: Appendix B.

Extracted information will include:

- publication year
- year of intervention
- study methodology
- study setting
- sample size (recruitment and study completion rates)
- study population (i.e., client specific factors including gender, age, offence type and risk of harm)
- details of the intervention and control conditions
- facilitator related factors, including type of facilitator (e.g., probation officer, psychologist), ratings of competence, training and experience
- treatment and implementation factors including number of therapy sessions, whether one‐to‐one or group setting, whether or not a manual is used and fidelity to the treatment protocol
- outcomes and times of measurement
- Risk ratios or detailed numerical data to calculate them

#### 3.3.5 Assessment of risk of bias in included studies

We will assess risk of bias using the Cochrane Risk of Bias tool (RoB 2) for randomised studies, and ROBINS‐I (Sterne, 2016) for non‐randomised studies. We will present detailed risk of bias data in a table describing each study with separate ratings for each risk of bias domain and an overall rating per study.

Risk of bias assessments will be undertaken by one review author, with a second researcher reviewing 25% of all assessments. A senior reviewer will assist this process, principally to resolve anything which it unclear or about which there is disagreement. All studies will be included in the subsequent review, but those judged to be high risk of bias will be subject to sensitivity analysis. Data permitting, risk of bias will also be coded and used as a moderator variable.

#### 3.3.6 Measures of treatment effect

We will calculate effect sizes for the final selection of studies, using a methodology which is appropriate for the included studies. We expect that the included studies will give both the overall numbers of participants, the numbers allocated to the treatment and control conditions, and the numbers reoffending in each condition (i.e., a binary outcome measure). We will use these data to calculate an odds ratio. If the 2×2 table data are not available, we will try to extract as much information as possible to calculate odds ratio. We will follow Polanin and Snilstveit (2016) if conversion from other effect sizes if necessary.

#### 3.3.7 Unit of analysis issues

The unit of analysis of interest for this review is the individual. However, careful consideration will be given to the unit of analysis of studies identified. Specifically, studies will be screened to identify instances where groups of individuals rather than individuals were randomised (i.e., cluster‐randomised trials), multiple intervention groups or multi‐arm trials have been used, and when multiple studies have used the same data source.

*Cluster‐randomised trials*. Unit‐of‐analysis errors (Whiting‐O'Keefe et al., 1984) occur when the unit of allocation differs from the unit of analysis. If clustering is ignored, and the analysis is conducted as if individuals were randomised (rather than the cluster), this will result in narrower confidence intervals, smaller p‐values, and ultimately, in the context of this review, these studies will receive more weight than appropriate (Higgins et al., 2023).

Where the appropriate cluster adjustments have not been made, we will follow the procedure to inflate the standard errors of the effect estimates, and thus reduce the size of each trial to its ‘effective sample size’ (Rao & Scott, 1992), as instructed in the Cochrane Handbook for Systematic Reviews of Interventions (Higgins et al., 2023). If clusters are similar in size, we will approximately calculate the design effect: $1+p\left(\bar{m}-1\right)$, where $\bar{m}$ is the average cluster size and p is the intra‐cluster correlation coefficient (ICC). Alternatively, if cluster size varies, Eldridge and Kerry (2012) suggest using the following to calculate the design effect:

$$1+p\left(m_{a}-1\right),\;\mathrm{where}\;m_{a}=\frac{\sum m_{i}^{2}}{\sum m_{i}},$$

where $m_{i}$ is the numbers of individuals in the ith cluster (Donner et al., 1981). An inflated standard error that accounts for clustering is then calculated by multiplying the standard error of the effect estimate by the square root of the design effect. The log risk ratio and inflated standard error can then be used in the methodological approach as stated in the data synthesis section. This will be followed by sensitivity analyses to investigate the robustness of our conclusions.

However, since the ICC is often not reported in studies, a common approach is to input a default ICC value based on empirical literature within the relevant field (Ahn et al., 2012; Higgins et al., 2023). For example, in conducting their Campbell Collaboration Systematic Review, Valdebenito et al. (2018) adopted this approach and used the ICC calculated by Ahn et al. (2012). Alternatively, where ICC data cannot be reasonably obtained (e.g., populations differ), Ahn et al. (2012) suggest estimating the ICC from the study itself using the variance/standard deviations and sample sizes typically reported for the treatment and intervention groups. This is the method we will adopt.

*Multiple intervention groups or multi‐arm trials*. As per Cochrane Handbook, where a study includes two or more intervention groups (e.g., different intensities of CBT programme delivery) against a control condition, we will combine intervention groups (if similar) into a single group to create a single pair‐wise comparison, providing all participants meet our eligibility criteria. If pooling is not appropriate (e.g., multiple intervention groups contain the same participants), we will choose one of the remaining methods recommended in the Cochrane Handbook (the choice will be dependent on a study) to avoid effect size multiplicity and the introduction of statistical dependency (López‐López et al., 2018). Information on all the intervention groups will be mentioned in the characteristics of included studies table but only a detailed description of the groups used in the analysis will be provided (Higgins et al., 2023).

#### 3.3.8 Dealing with missing data

Where outcomes were measured but data is missing or insufficient to calculate an effect size, we will attempt to contact the study authors to obtain the missing data. If this does not yield the required information, then we will still include the study in in the review but will only describe the study in the description of included studies section of the review and summarise the study in the characteristics of included studies table(s).

#### 3.3.9 Assessment of heterogeneity

All analyses will include an assessment of statistical heterogeneity, which will be reported in the form of Cochran's *Q*, Tau squared ($\tau^{2}$) and the *I* ^2^ statistic.

#### 3.3.10 Assessment of reporting biases

As our search strategy includes grey literature, this should help to mitigate any publication bias which might be observed if we were to only include published studies (as published studies are likely to report larger than average effects; Borenstein et al., 2011). We will, however, undertake additional analysis to assess whether publication bias is likely to be a factor in our findings. This will include a funnel plot to determine whether the summary effects of the review are subject to publication bias, and if this appears to be the case, further tests (e.g., Trim and Fill – Duval & Tweedie, 2000) to determine a ‘best estimate of the unbiased effect size’ (Borenstein et al., 2011, p. 286).

#### 3.3.11 Data synthesis

All analyses will be undertaken using a random effects model (using inverse‐variance weighting) to calculate summary odds ratios. Random effects models are appropriate when constituent studies differ in terms of mixes of participants and interventions (Borenstein et al., 2011).

This review and subsequent meta‐analysis will be undertaken using the metafor package in R (Viechtbauer, 2010). If only one outcome measure and time‐period are extracted from each study, we will apply univariate meta‐analysis. Initially, we will carry out an initial omnibus test, which includes all covariates and tests whether they are unrelated to the effect sizes, to explore the overall effect of cognitive‐behavioural programme participation on recidivism across all studies included in the review. Meta‐regression and ANOVA analysis will be implemented to test significance of each covariate separately.

If more than one outcome measurement is extracted per any one study, we will aim to apply multivariate meta‐analysis. This will be subject to being able to obtain correlation between outcome measures – ‘Unfortunately, determining the covariance among the effect sizes in this situation requires knowledge of the correlation between the measures. In our experience, this is rarely reported in most areas of research, which severely limits the practical utility of this approach despite its theoretical and statistical advantages’ (Lipsey & Wilson, 2001). If multivariate meta‐analysis is not achievable, we will run a meta‐analysis for each outcome measurement separately (e.g., re‐arrest, re‐conviction).

We will report overall weighted mean effects across the included studies with their relevant confidence intervals. Syntheses will also be presented in forest plots. We will provide effect sizes for all studies.

#### 3.3.12 Subgroup analysis and investigation of heterogeneity

Further to our initial omnibus analyses we will carry out separate subgroup analyses to explore any observed heterogeneity between the included studies. These analyses will focus on the study and measurement characteristics, and the implementation factors which we wish to understand (objective 2). They will be based on the following, assuming sufficient data of the required quality can be extracted:

– Recidivism measure (e.g., rearrest, reconviction, reincarceration)
– Source of outcome measure (e.g., administrative vs self report)
– Measurement duration (e.g., <12, 12, 18, 24, 36 months)
– Treatment (e.g., type, programme duration, contact hours per week)
– Proportion of intervention time that is constitutes a cognitive‐behavioural component (e.g., 100%, 50% to <100%, unknown %).
– Participant characteristics (e.g., gender, age, risk)
– Implementation factors (staff competence/experience, structured/manualised approach)
– Study location (USA, non‐USA)
– Study design (randomised, non‐randomised)
– Risk of bias (low, medium, high)

To test for any time‐variant effects of treatment (and potential interactions with implementation factors (objectives 3 and 4) we will undertake regression analyses similar to those described in Johnsen and Friborg (2015). In essence, these investigate the effect of study year as a predictor of recidivism in the meta‐analysis. We will use both linear and non‐linear meta‐regressions to build specifically on the findings and recommendations of Friborg and Johnsen (2017). If study year is not available in the published paper, we will contact the study authors to request this information. If we do not receive a response, we will choose the closest available year (e.g., publication year, or year of reporting for unpublished studies). In addition, we will investigate any hypothesised interaction effects between moderators (e.g., therapist competence/experience, manualised delivery as well as study design) and time. To triangulate our findings and to illustrate the effects of time, we will provide graphical examples using cumulative meta‐analysis (Leimu & Koricheva, 2004).

#### 3.3.13 Sensitivity analysis

We will undertake sensitivity analysis to determine the effect on our overall findings due to:

– Studies with effect sizes which have been converted from continuous to binary;
– Studies with high risk of bias;
– Studies where data has been imputed or other uncertain decisions or assumptions have been made about a study.

Sensitivity analyses will be undertaken by repeating the meta‐analyses, omitting in turn, each of the groups of studies described above, to determine their effect on overall findings.

## CONTRIBUTIONS OF AUTHORS

The authors will be responsible for the review as follows:

- Content: Chris Fox, Shadd Maruna, Will Cook, Anton Roberts
- Systematic review methods: Will Cook, Elizabeth Eggins, Chris Fox
- Statistical analysis: Karolina Krzemieniewska‐Nandwani, Will Cook
- Information retrieval: Elizabeth Eggins, Anton Roberts

## DECLARATIONS OF INTEREST

No potential conflicts of interest.

## PRELIMINARY TIMEFRAME

Approximate date for submission of the systematic review: September 2024.

## PLANS FOR UPDATING THIS REVIEW

Subject to identifying further funding we will update the review every 5 years.

## SOURCES OF SUPPORT

### Internal sources

No internal sources of support provided.

### External sources

The review will be funded by The Nuffield Foundation, and forms part of a wider project to understand the effectiveness of CBT in criminal justice.

## Supporting information

Supporting information.
