# Supplementary material for: Revisiting the effectiveness of cognitive‐behavioural therapy for reducing reoffending in the criminal justice system: A systematic review
Source: Campbell Syst Rev. 2024 Jul 31;20(3):e1425. doi: 10.1002/cl2.1425 (PMC11289900; doi:10.1002/cl2.1425)
Supplement: Supplementary file 1 — Supporting information. [file CL2-20-e1425-s001.docx]

Appendix A: Search framework and example search string

Template search structure

| **Line** | **Terms and Syntax** | **Search Fields** |
| --- | --- | --- |
| 1 | (anger N3 control*) OR (anger* N3 manage*) OR "automatic thought*" OR "behavio* analysis" OR "behavior* experiment*" OR "behaviour* experiment*" OR conditioned OR conditioning OR "core belief*" OR denseniti* OR "emotion* control*"or "emotional self-regulation" OR (emotion* N3 regulat*) OR exposure OR "guided imagery" OR "hassle log*" OR "intrusive thought*" OR operant OR reinforc* OR reward* OR resociali* OR schema* OR "self-statement logs" OR "social learning" OR "socratic question*" | TI, AB, KW, SU (Indexing / Controlled Vocab) |
| 2 | (cogniti* N3 behavio*) | TI, AB, KW, SU (Indexing / Controlled Vocab) |
| 3 | (think* N3 (change* OR distort* OR dysfunct* OR interven* OR maladaptive OR manage* OR model* OR modif* OR monitor* OR program* OR project* OR reform* OR restructur* OR skill* OR therap* OR train* OR treat*)) | Ti, AB, KW, SU (Indexing / Controlled Vocab) |
| 4 | (thought* N3 (change* OR distort* OR dysfunct* OR interven* OR maladaptive OR manage* OR model* OR modif* OR monitor* OR program* OR project* OR reform* OR restructur* OR skill* OR therap* OR train* OR treat*)) | TI, AB, KW, SU (Indexing / Controlled Vocab) |
| 5 | (cogniti* N3 (change* OR distort* OR dysfunct* OR interven* OR maladaptive OR manage* OR model* OR modif* OR monitor* OR program* OR project* OR reform* OR restructur* OR skill* OR therap* OR train* OR treat*)) | Ti, AB, KW, SU (Indexing / Controlled Vocab) |
| 6 | (behavio* N3 (change* OR distort* OR dysfunct* OR interven* OR maladaptive OR manage* OR model* OR modif* OR monitor* OR program* OR project* OR reform* OR restructur* OR skill* OR therap* OR train* OR treat*)) | TI, AB, KW, SU (Indexing / Controlled Vocab) |
| 7 | "Acceptance and Commitment Therapy" OR "aggression replacement training" OR BBR OR BRAVE OR "breaking free" OR "building better relationships" OR "bureau rehabilitation and values enhancement" OR C2C OR CBT OR COVAID OR "challenge to change" OR "control of violence for angry impulsive drinkers" OR DBT OR "Dialectical Behaviour* Therapy" OR "dialectical behavior* therapy" OR "Insight Prison Project" OR IPP OR MBCT OR metacognitive OR mindfulness OR "moral reconation" OR MRT OR "mental health step down" OR "rational emotive" OR REBT OR "reasoning and rehabilitation" OR "residential drug abuse program" OR RDAP OR "second wave" OR "seeking safety" OR "social skill* train*" OR SOTP OR SOTP-NR OR SOTP-R OR "steps toward awareness, growth and emotional strength" OR STAGES OR "Think First" OR "thinking for a change" OR T4C OR T4AC OR TSP OR "third wave" OR "therap* educat*" | TI, AB, KW, SU (Indexing / Controlled Vocab) |
| 8 | 1 OR 2 OR 3 OR 4 OR 5 OR 6 OR 7 | TI, AB, KW, SU (Indexing / Controlled Vocab) |
| 9 | arrest* OR convict* OR correctional OR crime* OR criminal* OR custod* OR delinquin* OR detain* OR forensic* OR gaol* OR imprison* OR incarcerat* OR inmate* OR jail* OR (juvenile* N3 justice) OR offend* OR offence* OR penal* OR penitentiar* OR prison* OR probat* OR re-arrest* OR re-convict* OR re-incarcerat* OR re-offen* OR rearrest* OR recidiv* OR reconvict* OR rehabilit* OR reincarcerat* OR relaps* OR reoffen* OR violat* | TI, AB, KW, SU (Indexing / Controlled Vocab) |
| 10 | evaluat* OR experiment* OR trial* OR RCT OR random* OR quasi* OR QED OR "difference-in-difference*" OR "difference in difference*" OR match* OR propensity OR psm OR "regression discontinuity" OR RDD OR effica* OR "treat* group*" OR "treat* condition*" OR "treat* participant*" OR "control* group*" OR "control* condition*" OR "control* participant*" | TI, AB, KW, SU (Indexing / Controlled Vocab) |
| 11 | 8 AND 9 AND 10 | TI, AB, KW, SU (Indexing / Controlled Vocab) |
| 12 | Limit 11 to English and 1965 – Current | |

Example search

**APA PsycInfo <1806 to November Week 4 2023>, via Ovid**

| **Line** | **Syntax** | **N Results** |
| --- | --- | --- |
| 1 | (anger N3 control* or anger* N3 manage* or "automatic thought*" or "behavio* analysis" or "behavior* experiment*" or "behaviour* experiment*" or conditoned or conditioning or "core belief*" or denseniti* or "emotion* control*or emotional self-regulation" or emotion* N3 regulat* or exposure or "guided imagery" or "hassle log*" or "intrusive thought*" or operant or reinforc* or reward* or resociali* or schema* or "self-statement logs" or "social learning" or "socratic question*").ab,id,ot,ti. | 374291 |
| 2 | (cogniti* adj3 behavio*).ab,id,ot,ti. | 83900 |
| 3 | (behavio* adj3 (change* or distort* or dysfunct* or interven* or maladaptive or manage* or model* or modif* or monitor* or program* or project* or reform* or restructur* or skill* or therap* or train* or treat*)).ab,id,ot,ti. | 210284 |
| 4 | (cogniti* adj3 (change* or distort* or dysfunct* or interven* or maladaptive or manage* or model* or modif* or monitor* or program* or project* or reform* or restructur* or skill* or therap* or train* or treat*)).ab,id,ot,ti. | 123655 |
| 5 | (think* adj3 (change* or distort* or dysfunct* or interven* or maladaptive or manage* or model* or modif* or monitor* or program* or project* or reform* or restructur* or skill* or therap* or train* or treat*)).ab,id,ot,ti. | 11919 |
| 6 | (thought* adj3 (change* or distort* or dysfunct* or interven* or maladaptive or manage* or model* or modif* or monitor* or program* or project* or reform* or restructur* or skill* or therap* or train* or treat*)).ab,id,ot,ti. | 7151 |
| 7 | ("acceptance and commitment therapy" or "aggression replacement training" or BBR or BRAVE or "breaking free" or "building better relationships" or "bureau rehabilitation and values enhancement" or C2C or CBT or COVAID or "challenge to change" or "control of violence for angry impulsive drinkers" or DBT or "dialectical behaviour* therapy" or "dialectical behavior* therapy" or "Insight Prison Project" or IPP or MBCT or metacognitive or mindfulness or "moral reconation" or MRT or "mental health step down" or "rational emotive" or REBT or "reasoning and rehabilitation" or "residential drug abuse program" or RDAP or "second wave" or "seeking safety" or "social skill* train*" or SOTP or SOTP-NR or SOTP-R or "steps toward awareness, growth and emotional strength" or STAGES or "Think First" or "thinking for a change" or T4C or T4AC or TSP or "third wave" or "therap* educat*").ab,id,ot,ti. | 147169 |
| 8 | exp "Acceptance and Commitment Therapy"/ or exp Behavior Therapy/ or exp Behavior Change/ or exp Cognitive Therapy/ or exp Cognitive Behavior Therapy/ or exp Dialectical Behavior Therapy/ or exp Exposure Therapy/ or exp Guided Imagery/ or exp Metacognitive Therapy/ or exp Mindfulness/ or exp Rational Emotive Behavior Therapy/ or exp Social Skills Training/ or exp Social Learning/ or exp Systematic Desensitization Therapy/ | 144642 |
| 9 | Anger Control/ or Behavior Analysis/ or Cognition/ or Cognitions/ or Cognitive Processes/ or Conditioning/ or Emotional Control/ or Emotional Regulation/ or Intrusive Thoughts/ or Negative Emotions/ or Reinforcement/ or Rewards/ or Schema/ | 200795 |
| 10 | 1 or 2 or 3 or 4 or 5 or 6 or 7 or 8 or 9 | 919258 |
| 11 | (arrest* or convict* or correctional or crime* or criminal* or custod* or delinquin* or detain* or forensic* or gaol* or imprison* or incarcerat* or inmate* or jail* or juvenile* N3 justice or offend* or offence* or penal* or penitentiar* or prison* or probat* or re-arrest* or re-convict* or re-incarcerat* or re-offen* or rearrest* or recidiv* or reconvict* or rehabilit* or reincarcerat* or relaps* or reoffen* or violat*).ab,id,ot,ti. | 295678 |
| 12 | exp Correctional Institutions/ or exp Correctional Psychology/ or exp Criminal Behavior/ or exp Criminal Justice/ or exp Criminal Offenders/ or exp Crime Prevention/ or exp Forensic Psychology/ or exp Incarcerated/ or exp Juvenile Justice/ or exp Juvenile Delinquency/ or exp Mentally Ill Offenders/ or exp Prisons/ or exp Reformatories/ | 80062 |
| 13 | 11 or 12 | 307558 |
| 14 | (evaluat* or experiment* or trial* or RCT or random* or quasi* or QED or "difference-in-difference*" or "difference in difference*" or match* or propensity or psm or "regression discontinuity" or RDD or effica* or "treat* group*" or "treat* condition*" or "treat* participant*" or "control* group*" or "control* condition*" or "control* participant*").ab,id,ot,ti. | 1553969 |
| 15 | exp Evaluation/ or exp Forensic Evaluation/ or exp Mental Health Program Evaluation/ or exp Program Evaluation/ or exp Psychiatric Evaluation/ or exp Randomized Controlled Trials/ or exp Treatment Effectiveness Evaluation/ or exp Treatment Outcomes/ | 276530 |
| 16 | 14 or 15 | 1686249 |
| 17 | 10 and 13 and 16 | 22671 |
| 18 | limit 17 to (human and english language and yr="1965 -Current") | 19197 |
| 19 | limit 18 to "remove medline records" | 10454 |

Appendix B: Data extraction form

|  |  |
| --- | --- |
| Bibliographic Information | |
| Authors | FREE TEXT |
| Title | FREE TEXT |
| Year | FREE TEXT |
| Source | FREE TEXT |
| Type | 1. Journal Article 2. Dissertation/Thesis 3. Report 4. Other |
| Abstract | FREE TEXT |
| Study description |  |
| Study undertaken in (country) | FREE TEXT |
| Study design | 1. Randomised 2. Non-randomised |
| Age of participants | 1. Adult 2. Juvenile 3. Both adult and juvenile |
| Gender *% (actual number)* | 1. Male 2. Female 3. Male and female |
| Participant risk | 1. High 2. Medium 3. Low |
| Setting | 1. Custody 2. Community 3. Custody and community |
| Intervention classification | 1. First wave CBT 2. Second wave CBT 3. Third wave CBT |
| *If third wave CBT* | 1. Acceptance and Commitment Therapy (ACT) 2. Dialectical Behaviour Therapy (DBT) 3. Mindfulness-Based Cognitive Therapy (MCBT) 4. Metacognitive Therapy (MCT) |
| Intervention components | 1. Cognitive restructuring 2. Cognitive skills training |
| Comparison (control) | 1. Treatment as usual   2. usual care or supervision’3. No treatment  4. Waitlist |
| Comparison intervention | FREE TEXT |
| Delivery mode | 1. Face-to-face 2. Online 3. App |
| Delivery format | 1. Group 2. Individual |
| Manualised | Y/N |
| Staff training level |  |
| Duration |  |
| Frequency (per month) |  |
| Contact hours (week) |  |
| Proportion of delivery time with a cognitive-behavioural component | 100%  50%-<100%  Unknown |
| Outcome measure (separate for each outcome) |  |
| Outcome source | 1. Administrative 2. Self report |
| Recidivism outcome measure | 1. Rearrest 2. Reconviction 3. Reincarceration |
| Recidivism outcome measure time | 1. <12 months 2. 12 months 3. 24 months 4. 36 months 5. Other |
| Other outcome(s) | FREE TEXT |
| Adverse outcome(s) | FREE TEXT |
| Total sample size | FREE TEXT |
| Size of treated group | FREE TEXT |
| Size of control group | FREE TEXT |
| Treated group reoffending (n) | FREE TEXT |
| Control group reoffending (n) | FREE TEXT |
| Author’s effect size | FREE TEXT |
| Author’s primary finding | FREE TEXT (i.e. quantified interpretation of effect size) |
| Treated group reoffending (%) | FREE TEXT |
| Control group reoffending (%) | FREE TEXT |
|  |  |
|  |  |
|  |  |
|  |  |
|  |  |
| Risk of Bias | As per RoB 2 and ROBINS-I |

Appendix C: Typology of CBT Waves

| Wave | Description | Examples |
| --- | --- | --- |
| 1 | Wave 1 (BT) behavioural therapy, originated as a more ‘empirical’ response to Freudian psychoanalysis as a treatment paradigm. Developed in a lab setting, it was associated with figures such as Watson and BF Skinner, and techniques of behavioural conditioning. With thinkers such as Aaron T Beck and Albert Ellis in the 1960s, there was a move towards the recognition of internal mental events, that behaviour was more complex than conditioned responses (Blackwell, Simon & Heidenreich, 2021). | Operant and classical forms of conditioning, skills training, and the exposure techniques often utilised when treating anxiety related disorders. |
| 2 | This second wave of understanding acknowledges and focuses on the role of cognitive processes such as cognitive restructuring, automatic thoughts, cognitive filters, cognitive distortions, and beliefs. In the treatment of mental disorders and creating lasting changing of behaviors (Brown, Gaudiano, & Miller, 2011). | Therapeutic examples of are rational emotive therapy (REBT), developed by Albert Ellis and Cognitive therapy Aaron Beck |
| 3 | Wave 3 grew from an acceptance that wave 1 and 2 still failed to acknowledge the complexity of patients and certain disorders e.g. personality disorders, therefore some CBT practitioners called for more of a focus on wider context and life goals- it attempts to be more holistic in its approach (Hofmann, Sawyer & Fang, 2010). It is associated with terms such as acceptance, curiosity, dialectics and takes an interest in human values. | Modern CBT interventions which include these additions are acceptance and commitment therapy, dialectical behavior therapy, mindfulness and acceptance cognitive therapy, and metacognitive therapy. |
